# Supplementary material for: Fecal shedding of Salmonella spp., Clostridium perfringens, and Clostridioides difficile in dogs fed raw meat-based diets in Brazil and their owners’ motivation
Source: PLoS One. 2020 Apr 14;15(4):e0231275. doi: 10.1371/journal.pone.0231275 (PMC7156072; doi:10.1371/journal.pone.0231275)
Supplement: S1 File — (DOCX) [file pone.0231275.s001.docx]

| **Questions for all participants (n=412)** |
| --- |

- What kind of feed do you provide to you dog?

| **Options** | **Answers (%)** |
| --- | --- |
| Exclusively commercial dry feed | 246 (59.7) |
| Exclusively RMBD | 166 (40.3) |
| Mixed or other diet* | Not included |
|  | 412 (100%) |

* No further questions were considered for this group.

- Did your dog present diarrhea in the last six months?

| **Options** | **RMBD-fed dogs** | **Dry feed-fed dogs** | **Total** |
| --- | --- | --- | --- |
| Yes | 53 (31.9) | 61 (24.8) | 114 (27.7) |
| No | 113 (68.1) | 185 (75.2) | 298 (72.3) |
| Total | 166 (100) | 246 (100) | 412 (100) |

| **Questions for those who fed their dogs exclusively dry feed (n=246)** |
| --- |

- Do you consider the possibility of fed your dog RMBD in the future?

| **Options** | **Answers (%)** |
| --- | --- |
| Yes | 91 (37) |
| No | 145 (58.9) |
| I do not know what RMBD is | 10 (4.1) |
|  | 246 (100) |

| **Questions exclusively for those who fed their dogs RMBD (n=166)** |
| --- |

- What is the main reason for you to adopt RMBD?

| **Options** | **Answers (%)** |
| --- | --- |
| “More natural for the animal” | 115 (69.3) |
| “It is healthier than dry feed” | 31 (18.7) |
| “Bad acceptance of dry feed” | 16 (9.6) |
| Other reason | 4 (2.4) |
|  | 166 (100) |

- How long since the adoption of RMBD?

| **Options** | **Answers (%)** |
| --- | --- |
| More than one year ago | 59 (35.5) |
| Less than one year ago | 107 (64.5%) |
|  | 166 (100) |

- Do you believe RMBD offers any health risk for you?

| **Options** | **Answers (%)** |
| --- | --- |
| No | 164 (98.8) |
| Yes | 2 (1.2) |
|  | 166 (100) |

- Do you believe RMBD offers any health risk for your dog?

| **Options** | **Answers (%)** |
| --- | --- |
| No | 146 (87.9) |
| Yes | 20 (12.1) |
|  | 166 (100) |

- Please mark if the following people are living in contact with dogs fed RMBD

| **Options** | **Answers (%)^*^** |
| --- | --- |
| Elderly (≥65 years) | 34 (20.5) |
| Children (<5 years) | 13 (7.3) |
| Immunocompromised individuals | 5 (3) |
| Pregnant women | 3 (1.8) |

* Percentage out of 166 respondents.
