# Supplementary material for: Fecal shedding of Salmonella spp., Clostridium perfringens, and Clostridioides difficile in dogs fed raw meat-based diets in Brazil and their owners’ motivation
Source: PLoS One. 2020 Apr 14;15(4):e0231275. doi: 10.1371/journal.pone.0231275 (PMC7156072; doi:10.1371/journal.pone.0231275)
Supplement: S2 File — (DOCX) [file pone.0231275.s002.docx]

| **Perguntas realizadas para todos participantes (n=412)** |
| --- |

- Que tipo de dieta você fornece para seu cão?

| **Opções** | **Respostas (%)** |
| --- | --- |
| Exclusivamente ração comercial seca | 246 (59,7) |
| Alimentação crua (RMBD) | 166 (40,3) |
| Outras dietas* | Não incluídos |
|  | 412 (100%) |

* Nenhuma pergunta adicional foi realizada para esse grupo.

- O seu cão apresentou diarreia nos últimos seis meses?

| **Opções** | **Cães alimentados com RMBD** | **Alimentação comercial seca** | **Total** |
| --- | --- | --- | --- |
| Sim | 53 (31,9) | 61 (24,8) | 114 (27,7) |
| Não | 113 (68,1) | 185 (75,2) | 298 (72,3) |
| Total | 166 (100) | 246 (100) | 412 (100) |

| **Questões destinadas apenas a tutores que alegaram fornecer exclusivamente ração comercial seca (n=246)** |
| --- |

- Você considera a possibilidade de fornecer RMBD para seu cão no futuro?

| **Opções** | **Respostas (%)** |
| --- | --- |
| Sim | 91 (37) |
| Não | 145 (58,9) |
| Desconheço RMBD | 10 (4,1) |
|  | 246 (100) |

| **Questões destinadas apenas a tutores que alegaram fornecer exclusivamente RMBD (n=166)** |
| --- |

- Qual sua principal razão para a adoção de RMBD?

| **Opções** | **Respostas (%)** |
| --- | --- |
| “É mais natural para o animal” | 115 (69,3) |
| “É mais saudável que ração comercial seca” | 31 (18,7) |
| “Baixa aceitação da ração comercial seca” | 16 (9,6) |
| Outras razões | 4 (2,4) |
|  | 166 (100) |

- Há quanto tempo você adotou RMBD para seu animal?

| **Opções** | **Respostas (%)** |
| --- | --- |
| Há mais de um ano | 59 (35,5) |
| Há menos de um ano | 107 (64,5%) |
|  | 166 (100) |

- Você acredita que a RMBD oferece riscos para você?

| **Opções** | **Respostas (%)** |
| --- | --- |
| Não | 164 (98,8) |
| Sim | 2 (1,2) |
|  | 166 (100) |

- Você acredita que a RMBD oferece riscos para seu cão?

| **Opções** | **Respostas (%)** |
| --- | --- |
| Não | 146 (87,9) |
| Sim | 20 (12,1) |
|  | 166 (100) |

- Marque caso pessoas que se encaixam nos seguintes perfis vivam em contato com cães alimentados com RMBD

| **Opções** | **Respostas (%)^*^** |
| --- | --- |
| Idosos (≥65 years) | 34 (20,5) |
| Crianças (<5 years) | 13 (7,3) |
| Indivíduos imunocomprometidos | 5 (3) |
| Grávidas | 3 (1,8) |

* Porcentual calculado considerando 166 respondentes.
